# The Circadian Protein PER1 Modulates the Cellular Response to Anticancer Treatments

**DOI:** 10.3390/ijms22062974

**Published:** 2021-03-15

**Authors:** Marina Maria Bellet, Claudia Stincardini, Claudio Costantini, Marco Gargaro, Stefania Pieroni, Marilena Castelli, Danilo Piobbico, Paolo Sassone-Corsi, Maria Agnese Della-Fazia, Luigina Romani, Giuseppe Servillo

**Affiliations:** 1 Department of Medicine and Surgery, University of Perugia, 06132 Perugia, Italy; claudiastincardini@gmail.com (C.S.); costacla76@gmail.com (C.C.); marco.gargaro@unipg.it (M.G.); stefania.pieroni@collaboratori.unipg.it (S.P.); marilena.castelli@collaboratori.unipg.it (M.C.); danilo.piobbico@collaboratori.unipg.it (D.P.); mariaagnese.dellafazia@unipg.it (M.A.D.-F.); luigina.romani@unipg.it (L.R.); 2 Department of Biological Chemistry, Center for Epigenetics and Metabolism, U1233 INSERM, University of California, Irvine, CA 92617, USA; psc@uci.edu

**Keywords:** circadian rhythm, period, p53, chronotherapy

## Abstract

The circadian clock driven by the daily light–dark and temperature cycles of the environment regulates fundamental physiological processes and perturbations of these sophisticated mechanisms may result in pathological conditions, including cancer. While experimental evidence is building up to unravel the link between circadian rhythms and tumorigenesis, it is becoming increasingly apparent that the response to antitumor agents is similarly dependent on the circadian clock, given the dependence of each drug on the circadian regulation of cell cycle, DNA repair and apoptosis. However, the molecular mechanisms that link the circadian machinery to the action of anticancer treatments is still poorly understood, thus limiting the application of circadian rhythms-driven pharmacological therapy, or chronotherapy, in the clinical practice. Herein, we demonstrate the circadian protein period 1 (PER1) and the tumor suppressor p53 negatively cross-regulate each other’s expression and activity to modulate the sensitivity of cancer cells to anticancer treatments. Specifically, PER1 physically interacts with p53 to reduce its stability and impair its transcriptional activity, while p53 represses the transcription of PER1. Functionally, we could show that PER1 reduced the sensitivity of cancer cells to drug-induced apoptosis, both in vitro and in vivo in NOD scid gamma (NSG) mice xenotransplanted with a lung cancer cell line. Therefore, our results emphasize the importance of understanding the relationship between the circadian clock and tumor regulatory proteins as the basis for the future development of cancer chronotherapy.

## 1. Introduction

Organisms have evolved to adapt their behavior and physiology to the daily light-dark and temperature cycles of the environment. This circadian rhythmicity is guaranteed by the presence of circadian clocks, collectively indicating central and peripheral endogenous systems that synchronize the organismal physiology to the environmental changes and optimize overall fitness [1]. Cell-autonomous and interlocking transcription-translation feedback loops govern the basic mechanism of circadian clock functioning [1]. In mammals, the main loop is based on two basic helix–loop–helix/PER–ARNT–SIM (bHLH-PAS) transcription factors, CLOCK and BMAL1, that heterodimerize to drive the rhythmic expression of period (Per) and cryptochrome (Cry) genes. In turn, the PER/CRY protein complexes inactivate CLOCK/BMAL1 (C/B), thus closing the circuit [2]. Disruption of these fine-tuning processes may reduce the fitness of the organism and increase the susceptibility to the development of pathological conditions, including cancer [3]. Indeed, multiple evidences have now been accumulated that link circadian clock with cancer. Epidemiological studies have shown that shift workers have an increased risk of cancer, and cancer patients share alterations in the expression of circadian genes [4]. In addition, circadian rhythm disruption is associated with tumors in animal models [5]. From a molecular level, the control that the circadian clock exerts over cellular checkpoints that prevent tumor initiation and progression is at basis of the link between circadian clock and cancer. For instance, the circadian clock controls proteins involved in the regulation of the cell cycle, the response to DNA damage, and DNA repair, a dysregulation of which leads to sustained proliferative signaling, evasion from growth suppressors, resistance to cell death, and replicative immortality, collectively representing recognized hallmarks of cancer [5]. The other side of the circadian rhythmicity is that anti-cancer therapies should take into account the periodic fluctuations of the physiological processes that regulate their pharmacokinetics and pharmacodynamics to increase the efficacy/safety profile [6]. Therefore, the circadian rhythms not only influence the process of tumorigenesis, but also the response of the tumor to therapeutic agents, with a view to a personalized medicine driven by circadian rhythms, or chronotherapy. 

In particular, the association of *Per* genes with cancer is increasingly being recognized [7] and their alterations in the expression have been associated with several types of human cancers [7]. Interestingly, *Per* genes are involved in the regulation of key processes in the development of cancer, including DNA damage response, cell cycle, proliferation and apoptosis [7]. One key regulator of many of these processes is the tumor suppressor p53 [8], whose mutation or deletion is found in more than 50% of human cancers. Moreover, the p53 pathway is thought to be functionally inactivated in the vast majority of cancers [9]. Understanding how tumor cells can become insensitive to p53 activation is therefore of major importance. 

It has been previously shown that p53 inhibits the expression of *Per2* gene, by preventing CLOCK/BMAL1 binding to the *Per2* promoter [10] and a similar mechanism also seems to apply to *Per1* [10], but the relationship between PER1 and p53 has never been investigated in depth. In the present study we have investigated the relationship between PER1 and p53 in the context of tumor susceptibility to chemotherapeutic drugs. We have also addressed the role of p53 in deregulation of the circadian gene *Per1*. We show that PER1 and p53 engage in a cross-regulatory interaction whereby p53 represses the expression of *Per1* and PER1, in turn, negatively regulate p53 activity. We also show how the PER1-p53 cross-talk regulates the susceptibility to chemotherapeutic drugs by influencing tumor cell apoptosis, thus opening up novel avenues for therapeutic intervention.

## 2. Results

### 2.1. PER1 Represses p53 Expression and Activity

**Figure 1:**
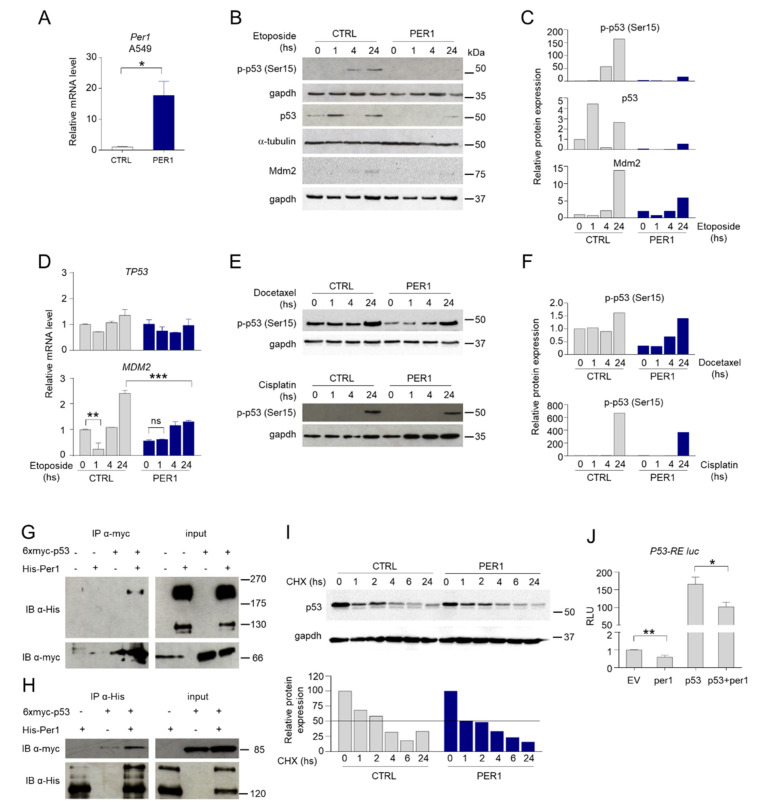
PER1 modulation of p53 expression and activity. (**A**) Control of PER1-overexpressing clones in A549 cells, evaluated by qPCR. A549 cells were transfected with PER1 or a control plasmid (CTRL, pEGFP N1) and positive clones were selected with G418 treatment. Bars represent average ± SD (n = 2). (*) *p* < 0.05. (**B**) Levels of p-p53 (Ser15), total p53 and Mdm2 following treatment with etoposide for the indicated time in A549 cells stably overexpressing PER1 or CTRL vector, evaluated by Western blotting (WB). Gapdh and α-tubulin were used as loading controls. (**C**) Densitometry for p-p53 (Ser15), total p53 and Mdm2 expression levels relative to (B). (**D**) *TP53* and *MDM2* gene expression levels in A549 cells stably overexpressing PER1 or CTRL vector treated as in 2B. Bars represent average ± SD (n = 2). (**) *p* < 0.01, (***) *p* < 0.001. (**E**) Levels of p-p53 (Ser15) following treatment with docetaxel or cisplatin for the indicated time in A549 cells stably overexpressing PER1 or CTRL vector, evaluated by WB. Gapdh was used as loading controls. (**F**) Densitometry for p-p53 (Ser15) expression levels relative to (E). (**G**–**H**) Immunoprecipitation (IP) between PER1 and p53. HEK-293 cells were cotransfected with myc-p53 and His-PER1. In (G) p53 was immunoprecipitated with anti-myc antibody. In (H) PER1 was immunoprecipitated with anti-His antibody. Co-immunoprecipitated proteins and total cell lysate (input) were analyzed by WB and revealed with anti-His and anti-myc antibodies. (**I**) Expression of p53 in A549 cells overexpressing PER1 or a control plasmid (CTRL) and treated with cycloheximide (CHX, 100 μM) for the indicated times. Gapdh was used as loading control. Densitometry for p53 expression levels is shown on the bottom. (**J**) Evaluation of the activation of p53-RE-Luc by luciferase assay in HEK-293 cells transfected with the indicated plasmids (ev = empty vector). Levels of expression are relative to the control transfected with empty vector, after normalization for transfection efficiency through beta-galattosidase assay. Bars represent average of RLU (relative luciferase units) ± SD (n = 3). (*) *p* < 0.05, (**) *p* < 0.01.

In order to study the relationship between p53 and PER1 in the context of cellular response to chemotherapeutic drugs, we stably overexpressed PER1 (PER1) or a control plasmid (CTRL) in the p53-sufficient human cancer A549 cells (Figure 1A). In these cells we evaluated p53 expression and activation upon treatment with etoposide, a drug generating DNA double-strand breaks which in turn activates p53 [11]. As shown, etoposide induced p53 phosphorylation at Ser-15 and increased p53 protein (Figure 1B–C), but not gene expression (Figure 1D) in control cells (Figure 1B,C). Interestingly, overexpression of PER1 almost completely abrogated etoposide-induced p53 protein expression and activation (Figure 1B,C), while *TP53* mRNA levels were not modified (Figure 1D), indicating that PER1 negatively regulated p53 at a protein level. 

To test the hypothesis that PER1 could modulate p53 activation following treatment with different cytotoxic drugs, we treated CTRL and PER1 A549 cells with other chemotherapeutics with different mechanisms of action, such as the microtubule disruptor docetaxel and the DNA-crosslinking platinum-based agent cisplatin. Similarly to cells treated with etoposide, p53 phosphorylation was reduced in PER1 A549 cells treated with docetaxel or cisplatin compared to CTRL cells (Figure 1E,F), thus confirming that PER1 interferes with p53 activation induced by different stimuli. 

Given the control of PER1 on p53 at the post-translational level, we analyzed a putative role played by MDM2, the most important negative regulator of p53 in controlling p53 stability [12]. We explored the possibility that PER1 overexpression could increase MDM2 transcription following etoposide treatment, therefore reducing p53 stability. Treatment with etoposide in CTRL cells induced down-regulation of MDM2 at early time points, as previously described [13], followed by a significant induction within 24 h (Figure 1D). PER1 overexpression was able to prevent the initial down-regulation of MDM2, while the level of induction at later time points was substantially lower (Figure 1D), leading to undetectable protein expression levels (Figure 1B,C). Thus, PER1 does not modulate p53 function by acting on MDM2, but we could hypothesize that PER1 directly regulate p53 function. Indeed, by transfecting A549 cells with Myc-tagged p53 and His-tagged PER1, we showed that the proteins co-immunoprecipitated (Figure 1G–H), indicating that p53 and PER1 can physically interact. Irrespective of the mechanism involved, p53 half-life was reduced in PER1 overexpressing cells (Figure 1I), suggesting that PER1 negatively regulated p53 stability and impaired its activation in response to DNA-inducing stimuli. 

The specific binding of p53 with PER1 prompted us to explore whether this interaction could be functionally relevant in modulating p53-driven transcriptional activation. To address this question we performed luciferase assays on transiently transfected cultured cells. Using a synthetic reporter vector carrying p53 responsive elements (p53-RE) fused to the luciferase gene, we demonstrated that PER1 overexpression was able to induce a modest but significant repression of the transactivation of p53-RE mediated by p53 (Figure 1J). All together, these results suggest an active role of PER1 in controlling p53 activation during anticancer treatments.

### 2.2. PER1 Controls Apoptosis Induced by Chemotherapy Drugs Through p53

**Figure 2:**
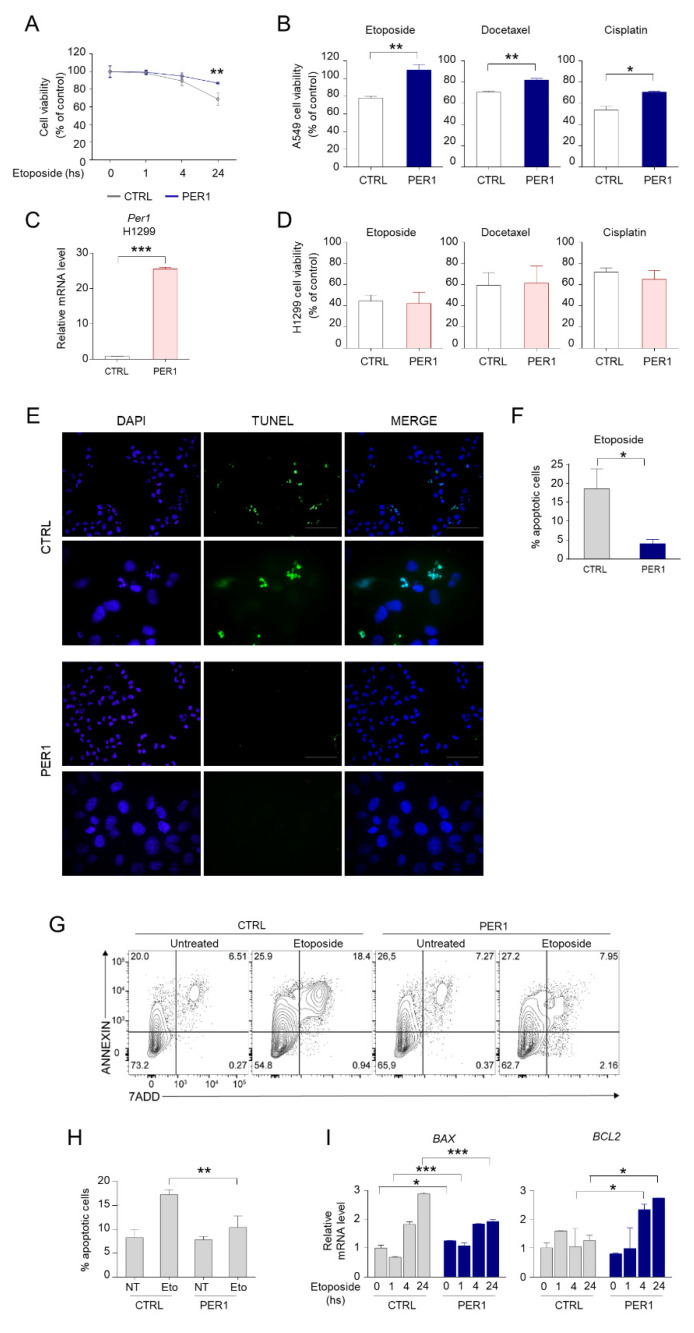
PER1 downregulation of apoptosis. (**A**) Cell viability following treatment with etoposide for the indicated time in A549 cells overexpressing PER1 or CTRL vector, measured by MTT assay and expressed as % of control untreated cells. Bars represent average ± SD (n = 3). (**) *p* < 0.01. (**B**) Cell viability following treatment with etoposide, docetaxel or cisplatin for 24 h in A549 cells overexpressing PER1 or CTRL vector, expressed as % of control untreated cells. Bars represent average ± SD (n = 3). (*) *p* < 0.05, (**) *p* < 0.01. (**C**) Control of PER1-overexpressing clones in H1299 cells, evaluated by qPCR. H1299 cells were transfected with PER1 or a control plasmid (CTRL, pEGFP N1) and positive clones were selected with G418 treatment. Bars represent average ± SD (n = 2). (***) *p* < 0.001. (**D**) Cell viability following treatment with etoposide, docetaxel or cisplatin for 24 h in H1299 cells overexpressing PER1 or CTRL vector, expressed as % of control untreated cells. Bars represent average ± SD (n = 3). (**E**) Representative image of TUNEL staining of A549 cells overexpressing PER1 or CTRL vector treated with etoposide. Nuclei were counterstained with DAPI. 40× and 100× magnification (scale bars, 100 and 50 μm, respectively). (**F**) Percentage of apoptotic cells in CTRL and PER1 cells obtained by TUNEL-positive cell count. Bars represents means ± SD (n = 5 fields of observation from three samples each group). (**G**) Measure of apoptotic cells in A549 cells overexpressing PER1 or CTRL untreated or treated with etoposide, analyzed by flow cytometry. (**H**) Percentage of apoptotic cells in CTRL and PER1 cells, obtained by flow cytometry analysis. Bars represents average ± SD (n = 3). (**I**) *BAX* and *BCL2* gene expression levels in A549 cells overexpressing PER1 or CTRL vector and treated with etoposide for the indicated time. Bars represent average ± SD (n = 2). (*) *p* < 0.05, (**) *p* < 0.01, (***) *p* < 0.001.

We next examined whether reduced p53 activation mediated by PER1 was associated to a functional defect of p53 downstream pathways. We observed that cell viability was increased in PER1 A549 cells, compared to CTRL cells, after 24 h of treatment with etoposide (Figure 2A–B). The protective effect of PER1 was also evident during treatment with other chemotherapeutic drugs, including docetaxel and cisplatin (Figure 2B). Interestingly, such protective action was prevented in the p53-defective H1299 cell line stably overexpressing PER1 (Figure 2C–D), indicating that the presence of p53 is required for the effects of PER1 to occur. We hypothesized that the increased cell survival after chemotherapy treatment in PER1 cells was associated to reduced p53-mediated apoptosis. To this purpose, we performed a TUNEL assay in A549 cells treated with etoposide and we confirmed the reduced number of apoptotic cells in A549 cells overexpressing PER1 compared to CTRL cells (Figure 2E–F). A FACS analysis in etoposide treated A549 cells further corroborates our findings. Annexin V staining revealed that PER1 overexpression resulted in protection from etoposide-induced apoptosis in these cells (Figure 2G–H). This was associated with differential regulation of the Bcl-2 family members, with down-regulation of the pro-apoptotic *BAX* and up-regulation of the pro-survival *BCL-2* genes in PER1 overexpressing cells following 24 h of treatment (Figure 2I).

### 2.3. p53 Represses the Transcription of Per1 gene

**Figure 3:**
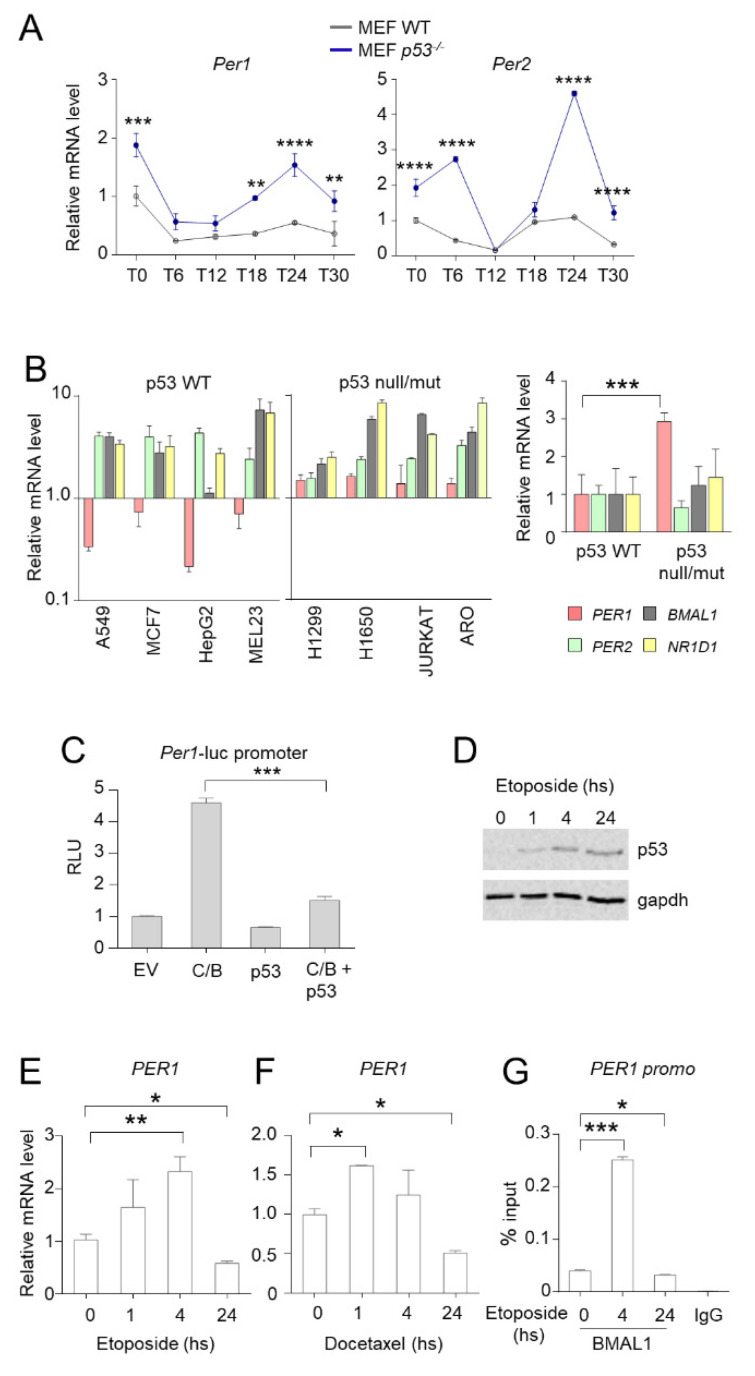
Control of *PER1* expression by p53. (**A**) Circadian expression of *Per1* and *Per2* in MEFs WT and p53^−/−^. Real-time PCR analysis was performed from RNAs prepared at the indicated times after serum shock synchronization. T0, unsynchronized cells. Bars represent average ± SD (n = 2). (**) *p* < 0.01, (***) *p* < 0.001, (****) *p* < 0.0001. (**B**) Q-PCR analysis of *PER1*, *PER2*, *BMAL1* and *NR1D1* from RNA samples prepared from different human cancer cell lines. Espression value in non-tumoral HEK-293 cells was set to 1. Bars represent average ± SD (n = 3). Right, average levels of expression of the same genes in p53 wild-type cell lines (A549, MCF7, HepG2, MEL23) versus p53 null/mut cell lines (H1299, H1650, JURKAT, ARO). Bars represent average ± SD. (***) *p* < 0.001. (**C**) Evaluation of the activation of *Per1* promoter by luciferase assay in HEK-293 cells transfected with the indicated plasmids (C/B = Clock/Bmal1; ev = empty vector). Levels of expression are relative to the control transfected with empty vector, after normalization for transfection efficiency through beta-galattosidase assay. Bars represent average of RLU (relative luciferase units) ± SD (n = 3). (***) *p* < 0.001. (**D**) Expression of p53 following treatment with etoposide for the indicated time in A549 cells, evaluated by WB. Gapdh was used as loading control. (**E**–**F**) *PER1* mRNA expression levels in A549 cells treated with etoposide (**E**) or docetaxel (**F**) for the indicated time. Bars represent average ± SD (n = 2). (*) *p* < 0.05, (**) *p* < 0.01. (**G**) ChIP from A549 cells treated with etoposide for the indicated time. Cells were collected after treatment, subjected to dual cross-link and ChIP with BMAL1 antibody or mouse IgG was performed. Primers for *PER1* promoter were used for qPCR. BMAL1 enrichment was shown as % of input. Bars represent average ± SD (n = 3). (*) *p* < 0.05, (***) *p* < 0.001.

Whereas PER1 has a role in controlling the activity of p53, then the activation of p53 could determine a modulation of the expression of PER1. To this purpose, we first evaluated the circadian expression of *Per* genes in wild-type and p53-deficient MEF cells (Figure 3A). In line with the suppressor activity of p53 on the expression of *Per* genes [10], we found an up-regulation of both *Per1* and *Per2* expression in the relative absence of p53 while the circadian rhythmicity was maintained (Figure 3A). To corroborate these findings in human cancer cells, we analyzed the expression of *PER1* and other clock genes (*PER2*, *BMAL1* and *NR1D1*) in different cancer cell lines with functional or defective p53. Compared with non-tumoral cell lines, the p53-sufficient cells (A549, MCF7, HepG2, MEL23) have reduced *PER1* expression levels, while *PER2*, *BMAL1* and *NR1D1* expression increased. On the contrary, in cell lines with deletion (H1299) or mutation (H1650, Jurkat, Aro) of p53, *PER1* expression was significantly increased, suggesting that the removal of p53 repression up-regulates the levels of *PER1* (Figure 3B). 

Since *Per1* is mainly regulated by CLOCK and BMAL1 at the transcriptional level, our hypothesis was that *Per1* repression induced by p53 could be due to impaired activation of *Per1* promoter by C/B. To prove that p53 negatively regulates the expression of *Per1* driven by CLOCK and BMAL1, a reporter constituted by the *Per1* gene promoter fused with the luciferase gene was ectopically expressed in HEK-293 cells by transient transfection. Co-expression of C/B resulted in activation of the *Per1* promoter, as previously reported [14]. Concomitant transfection of p53 has no effect on the basal *Per1* promoter activity, while it significantly reduced C/B-driven activation (Figure 3C). 

In order to determine whether *PER1* inhibition also occurred following anti-tumoral treatment, we evaluated the expression of *PER1* upon treatment of A549 cells with etoposide. As above described, etoposide treatment increased p53 stability (Figure 3D). Surprisingly, we observed that the expression of *PER1* was induced within 4 h of treatment, while it was significantly inhibited at longer time points (Figure 3E). A similar result was obtained in cells treated with docetaxel (Figure 3F). These results were further confirmed by using a chromatin immunoprecipitation approach, which demonstrated that BMAL1 is present at the promoter of *PER1* in unstimulated A549 cells, while its recruitment at the chromatin level is significantly enhanced upon 4 h of treatment with etoposide and subsequently inhibited at 24 h (Figure 3G). Thus, p53-mediated inhibition of *PER1* might take place during long lasting treatments. 

In conclusion, these results indicate that a cross-talk takes place between PER1 and p53, with p53 modulating the expression of *PER1*, and PER1 regulating the activation of p53, a feedback loop that may condition the susceptibility to a variety of chemotherapeutic drugs.

### 2.4. PER1 Reduces the Sensitivity to Chemotherapy Drugs In Vivo

**Figure 4:**
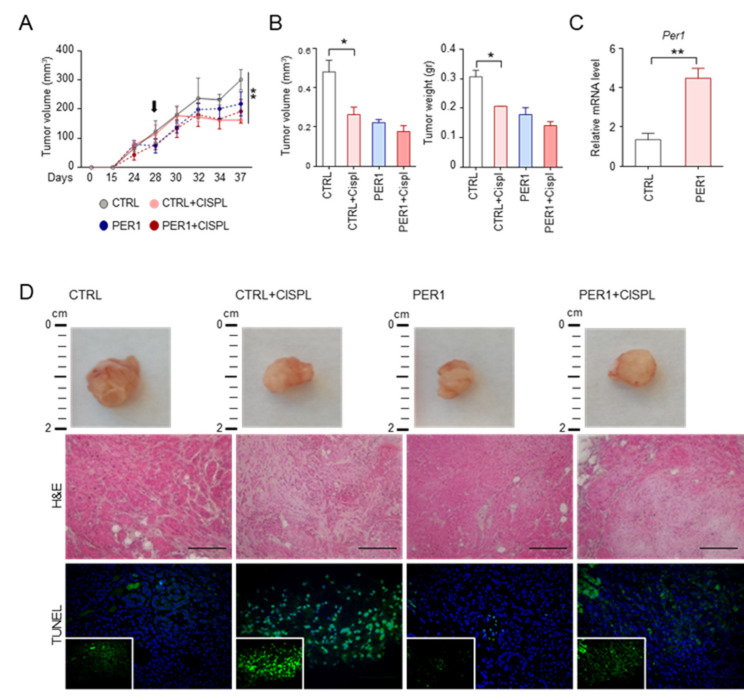
Resistance to treatment in PER1-overexpressing tumors. (**A**) Tumor volume over time of A549-GFP and A549-PER1 subcutaneous tumor xenografts in NSG mice treated with cisplatin. Black arrow: first day of treatment with cisplatin at the concentration of 5 mg/kg, repeated every 3 days for two weeks. Bars represent average ± SEM (n=4 mice). (**) *p* < 0.01. (**B**) Tumor volume and tumor weight after 15 days of treatment as in (A). Bars represent average ± SEM (n = 4 mice). (*) *p* < 0.05. (**C**) Control of *Per1* level of expression in A549-GFP (CTRL) and A549-PER1 (PER1) subcutaneous tumors, evaluated by qPCR. Bars represent average ± SD (n = 4 mice). (**D**) Representative images of gross histology, histology (H&E staining) and tumor cell death (TUNEL). 40× magnification (scale bars, 100 μm).

To demonstrate the relevance of the PER1-p53 cross-talk in the context of tumor cells growth and susceptibility to chemotherapy, the effect of PER1 in reducing the cellular response to anticancer treatments was evaluated in an in vivo setting. Specifically, NOD scid gamma (NSG) mice, which have a high susceptibility to xenotransplanted lung cancer cell lines [15] were subcutaneously inoculated with A549 PER1 or CTRL cells. Mice were treated at the indicated time with cisplatin, one of the most widely used first-line chemotherapy in non-small cell lung cancer treatment, to which A549 displayed a high susceptibility in vivo [16]. Tumor volume was evaluated during treatment (Figure 4A) and after mice sacrifice (Figure 4B). *Per1* overexpression in tumors was confirmed by qPCR (Figure 4C). In line with previous findings showing that PER1 expression suppresses growth of human cancer cells [17], PER1-overexpressing tumors were smaller than controls, as indicated by tumors volume and weight and by gross histology (Figure 4A,B,D). Interestingly, the treatment with cisplatin in mice inoculated with CTRL cells significantly reduced tumor growth, while promoting tumor cell death, as revealed by decreased cell density and increased grey area in tumor histology and by TUNEL assay (Figure 4D). Conversely, a reduced tumor growth-suppressing effect was observed in PER1-overexpressing tumors (Figure 4A,B,D). This result further demonstrate that the presence of high levels of PER1 is able to confer resistance to chemotherapy to lung cancer cells harboring wild type p53.

## 3. Discussion

In the last decades, a series of studies have supported the role of Period proteins as candidate tumor suppressors. In particular, the importance of PER1 has been highlighted by studies demonstrating that PER1 is downregulated in different types of human cancers and that its expression leads to growth suppression of human cancer cells [17,18,19,20]. At a molecular level, PER1 plays a direct role in the first steps of DNA damage response, by interacting and activating the cell cycle checkpoint proteins ATM and Chk2 [17]. Instead, the role of PER1 in the regulation of apoptosis is still not completely elucidated, since different studies reported both a pro- and an anti-apoptotic function of PER1 [17,21,22]. Consequently, PER1 could variably modulate the resistance of tumor cells against chemotherapy or radiation therapy. For example, it has been reported that PER1 sensitizes cancer cells to apoptosis induced by ionizing radiation, by suppressing p21-mediated cell cycle arrest via c-Myc induction [17]. Instead, other reports described that *Per1/2* KO mice displayed less cisplatin resistance compared to WT mice associated to increased p53 induction in a B16F10 melanoma mouse model [23]. An anti-apoptotic role of PER1 has been described in different human cancer cell lines [21,22]. Moreover, a downregulation of p53 signaling induced by overexpression of PER1 has been documented in a microarray analysis conducted in colangiocarcinoma cells [20].

The results presented in this study extend the previous findings on the role of PER1 in the cellular response to anticancer treatments, particularly focusing on its functional and molecular interaction with p53. Here, we demonstrated that increased levels of PER1 in A549 cells negatively modulates p53 signaling, possibly through a mechanism that involves a direct binding between PER1 and p53, a destabilization of p53 not mediated by Mdm2 and a repression of p53-mediated transcription. The repressive effect of PER1 on p53 results in reduced apoptosis and increased resistance to different anticancer agents, both in vitro and in vivo in xenograft models. Thus, PER1 level of expression in cancer cells could be importantly correlated to p53 level of activation.

**Figure 5:**
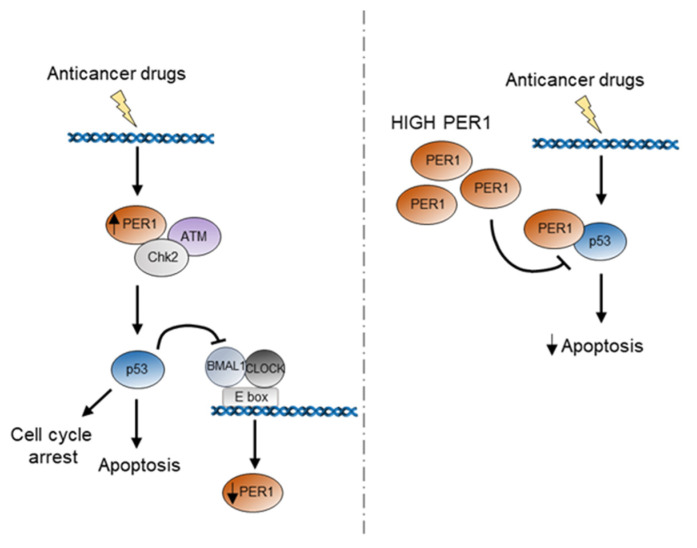
Schematic representation of the potential role of PER1 during p53 activation in response to anticancer treatment. In the first hours following anticancer treatment, PER1 is induced and takes part to the activation of the DNA damage response through binding and induction of the ATM-Chk2 checkpoint kinases. The subsequent activation of p53 determines a repression of PER1 expression. Indeed, high levels of PER1 reduce the stability of p53 and determine a repression of p53-mediated apoptosis, ultimately leading to reduced sensitivity of cancer cells to chemotherapy treatment.

A deregulation of circadian rhythms is a common feature of cancer cell lines and advanced-stage tumors, with the level of circadian disruption in human tumor tissues correlating with cancer prognosis and survival [5]. This deregulation has been variably associated to genetic alterations occurring in cancer cells, leading to activation of oncogenes (c-myc, K-RAS) or inhibition of tumor suppressor genes (p53), which are all involved in circadian rhythm dysregulation [10,24,25]. Here, we observed that, in murine cells lacking p53 and human cancer cell lines with mutation or deletion of p53, PER1 was expressed to high levels, compared to wild type cells. We also demonstrated that the ability of p53 to repress *PER1* transcription relies on a reduced binding of C/B to *PER1* promoter. This inverse relationship between p53 and PER1 levels was further examined during treatment with p53 activating agents. We observed that different anticancer treatments, while increasing and stabilizing p53 protein, reciprocally determine an initial rise in *PER1* expression, followed by a reduction as soon as p53 reaches the highest levels. Given the direct role of PER1 in activating the ATM-Chk2 complex [17], here we propose a model in which PER1 is initially required in the first steps of DNA damage response for the proper activation of the cell cycle checkpoint proteins. Subsequently, p53 stabilization and activation might be responsible of PER1 down-regulation (Figure 5). Therefore, an auto-regulatory feedback loop involving PER proteins and p53 might be operative in some experimental settings in which PER proteins controls p53 activation that, in turn, represses the transcription of Period genes, a circuit that may have implications not only in cancer initiation and progress, but also in the therapy of cancer. Furthermore, the relationship between PER1 and p53 could be more complex than imagined. Indeed, the two genes are closely located in human chromosome 17p13.1. Deletion of both genes is constantly present in case of somatic heterozygous deletions of chromosome 17p. In addition to p53, this region encodes over 300 genes, encoding many established or putative tumor suppressors, the suppression of which has been shown to cooperate with p53 to produce more aggressive diseases [26]. The exact role of PER1 in this scenario is all to be investigated. A translocation involving *PER1* gene has been described associated to occurrence of acute myeloid leukemia, and the inactivation of the gene has been associated to the pathogenesis of the disease [27]. Uncovering in full how PER1 and p53 are mutually regulated will be very important for a complete comprehension of the impact of this gene in cancer development and response to therapeutic strategies.

Whereas the reduced sensitivity of cancer cells to chemotherapeutics induced by PER1 is detrimental because it compromises treatment efficacy, this effect might instead confer protection from toxic side effects of cytotoxic treatments in non-tumor cells with an intact circadian clock. Indeed, the chrono-pharmacological studies conducted on mice over the last decades have revealed daily differences in toxicity for different drugs to target tissues, including the bone marrow and the gastrointestinal tract. Intriguingly, PER1 might contribute to this different susceptibility by conferring protection at the circadian time during which it is expressed the most. Future studies are needed in order to provide a better understanding of the role of PER1 in the cellular response to anticancer treatments in normal and tumor cells.

## 4. Materials and Methods

### 4.1. Plasmids

pCDNA3.1 V5-His mPer1 was provided by Dr. Steven Reppert. pCMV Sport2 mPer1 was a gift from Cheng Lee (Addgene plasmid # 16203; http://n2t.net/addgene (accessed on 25 January 2021): 16203; RRID:Addgene_16203) [28]. Plasmids expressing both β-galactosidase (pGL3-lacZ) for transfection control, and luciferase (luc) for luminometry-based expression, pGL3-mPer1-Luc promoter, were described previously [29]. N-terminal Myc-tagged plasmids Myc-CLOCK/pSG5, Flag-myc-BMAL1/pCS2+MT were previously described [30]. Plasmid encoding myc-p53 pCMV was provided by P.G. Pelicci, plasmid encoding p53-RE Luc was a gift of S. Soddu.

### 4.2. Reagents and Antibodies

Etoposide, docetaxel and cisplatin were from Sigma-Aldrich (St. Louis, Missouri, USA) and used at the indicated concentration. Antibodies against p53 (sc-263), Mdm2 (sc-965) and Bmal1 (sc-365645) were from Santa Cruz Biotechnology (Santa Cruz, California, USA), phosho-p53 (Ser15) (16G8) was from Cell Signaling Technology (Danvers, Massachusetts, USA), anti-β-tubulin, anti-Gapdh, anti-His and anti-myc were from Sigma-Aldrich.

### 4.3. Cell Culture and Circadian Synchronization

HEK 293T cells (Human Embryonic Kidney 293T, ATCC, Manassas, Virginia, USA) were maintained in Dulbecco’s modified Eagle medium (DMEM, 4.5 g/L glucose, Euroclone, Milan, Italy) supplemented with 10% fetal bovine serum (FBS), antibiotics, L-glutamine and non-essential amino acids (NEAA) (EuroClone) and cultured at 37 °C in 5% CO2. A549 and H1299 cells were grown in RPMI1640 (EuroClone) supplemented with 10% FBS, antibiotics, L-glutamine, and cultured at 37 °C in 5% CO_2_. All cell lines described in Figure 3B were grown in accordance with ATCC recommendations and tested for mycoplasma contamination. p53^−/−^ MEFs were a kind gift from P.G. Pelicci and maintained as described previously [31] For circadian experiments, after 2 h of 50% horse serum containing media serum shock, cells were incubated with serum-free medium for the indicated time.

### 4.4. Transient and Stable Transfection, Luciferase Assay and Cells Treatments

Cells were seeded in 24-well plates at a density of 7.5 × 10^4^ cells per well. Cells were transfected according to the manufacturer’s protocol. Lipofectamine^®^ LTX (Thermo Fisher Scientific, Waltham, Massachusetts, USA) was used to transfect A549 cells and Fugene^®^6 (Promega Corporation, Madison, WI, USA) was used to transfect HEK 293 and H1299 in accordance with the manufacturer’s instructions. Positive clones were selected with G418 treatment. Cell extracts were subjected to a luminometry-based luciferase assay, and luciferase activity was normalized by β-galactosidase activity. For p53 half-life, cells at 80% confluence were treated with cycloheximide (CHX 100 μM, Sigma-Aldrich) and analyzed by Western blotting (WB) at the indicated time points. Treatment with etoposide (5 μM, Sigma-Aldrich), docetaxel (100 ng/mL, Sigma-Aldrich) and cisplatin (10 μM, Sigma-Aldrich) were performed at the indicated time points.

### 4.5. Western Blotting (WB) and Immunoprecipitation (IP)

Protein extracts were denatured by adding Laemmli buffer (Tris/HCl at pH 6.8, 200 mM, SDS 8%, bromophenol blue 0.4%, glycerol 40% and β-mercaptoethanol 5%) and boiled for 5 min at 95 °C. Proteins were separated on polyacrylamide gel (Bio-Rad, Hercules, California, USA) and transferred by electroblotting onto nitrocellulose membranes (Bio-Rad). Blots were blocked in dry fat-free milk 5% in PBS 1× for 1 h and then incubated with primary antibody overnight (ON) at 4 °C. Detection was achieved using horseradish-peroxidase-conjugated secondary antibody (Bio-Rad) and visualized with ECL (GE Healthcare Life Sciences, Little Chalfont, UK). For the immunoprecipitation assay, cells were harvested 24 h after transfection, washed twice with cold phosphate buffered saline (PBS, Sigma-Aldrich) and lysed in RIPA buffer (Tris/HCl at pH 8.0, 50 mM, NaCl 150 mM, SDS 0.1%, sodium deoxycholate 1%, Triton X-100 1%), supplemented with protease inhibitor cocktail (PIC) and phenylmethylsulfonyl fluoride (PMSF) (Sigma-Aldrich). Lysates were sonicated 2 x 10 sec and cleared, and proteins were quantified by Bio-Rad assay (Bio-Rad). Lysates were incubated overnight at 4°C with the specific antibody, followed by 2 h of incubation with protein G- or protein A-Sepharose beads (GE Healthcare Lifescience, Little Chalfont, United Kingdom). Immune complexes were washed four times in washing buffer and boiled in 2× sample buffer. The proteins were separated by electrophoresis on SDS-PAGE and detected using specific antibodies. Protein signal intensities were quantified by densitometric analysis using Image J software.

### 4.6. RNA Extraction and Quantitative Real-Time PCR (qPCR)

For gene expression analysis by qPCR, total RNA was extracted with TRIzol Reagent (Thermo Fisher Scientific) and processed according to the instructions of the manufacturer. Reverse transcription was performed using 1 μg of input RNA from each sample with iScript RT Supermix (Bio-Rad Laboratories), and 4-fold diluted cDNA was used for each PCR reaction. For a 20 μL of PCR, 50 ng of cDNA was mixed with primers to final concentration of 150 nM and 10 μL of Brilliant SYBR^®^Green QPCR Master Mix and ROX (Agilent Technologies Inc., Santa Clara, California, USA) as reference dye. The reaction was first incubated at 95 °C for 3 min, followed by 40 cycles at 95 °C for 30 s and 60 °C for 1 min. qPCR was performed by monitoring in real-time the increase in fluorescence on an Mx3000P™Real-Time PCR detector system (Agilent Technologies). Results were analysed using the 2-DDCt method to calculate the relative level of each mRNA and expressed as a ratio relative to 18S rRNA, Beta-actin or Gapdh housekeeping genes. The following primers were used: 18S: forward CGGACACGGACAGGATTGACAG, reverse ATCGCTCCACCAACTAAGAACGG; Beta-actin: forward CACTCTTCCAGCCTTCCTTCC, reverse ACAGCACTGTGTTGGCGTAC; GAPDH: forward GTCAGTGGTGGACCTGACCT, reverse TGCTGTAGCCAAATTCGTTG; HPRT: forward CGAGATGTGATGAAGGAGATGGG, reverse GATGTAATCCAGCAGGTCAGCAA; Per1: forward CTGGCAATGGCAAGGACTC, reverse AGGAGGCTGTAGGCAATGG; Per2: forward CGCCTAGAATCCCTCCTGAGA, reverse CCACCGGCCTGTAGGATCT; PER1: forward CAGTGCTCCTGTTCCTGCATC, reverse CCCGCCAACTGCAGAATCT; PER2: forward AATGCCGATATGTTTGCGGT, reverse GCATCGCTGAAGGCATCTCT; BMAL1: forward CCAGAGGCCCCTAACTCCTC, reverse TGGTCTGCCATTGGATGATCT; NR1D1: forward CCGTGACCTTTCTCAGCATGA, reverse GACTGTCTGGTCCTTCACGTTG; PER1 promo: forward TGTCTCTCCCCTCCTCTCAA, reverse AGATACGCTGCGCCTCTTTA; TRP53: forward TTTGCGTGTGGAGTATTTGGATG, reverse CCAGTGTGATGATGGTGAGG; MDM2: forward GAATCATCGGACTCAGGTACATC, reverse TCTGTCTCACTAATTGCTCTCCT; BAX: forward CTGCAGAGGATGATTGCCG, reverse TGCCACTCGGAAAAAGACCT; BCL2: forward TCCCTCGCTGCACAAATACTC, reverse ACGACCCGATGGCCATAGA.

### 4.7. MTT Assay

Cells were seeded on 24-well plates at approximately 50% confluence. The following day, cells were treated with the indicated drugs or vehicle control. After 24 h medium was removed and cells were incubated with 1 mg/mL of 3-(4,5-dimethylthiazol-2-yl)-2,5-diphemyltetrazolium bromide (MTT, Sigma Aldrich) in PBS for 20 min at 37 °C. After carefully removing MTT solution, cells were resuspended in 300 µL of DMSO, and cell viability was measured on the Spark multimode microplate reader (Tecan Trading AG, Switzerland) by detecting absorbance at 570 nm.

### 4.8. Apoptosis Analysis

The study of apoptosis in cells was conducted by FACS analysis using the PE Annexin V Apoptosis Detection Kit (BD PharMingen, San Diego, CA) according to the manufacturer’s instructions. Briefly, 10^6^ cells treated with etoposide (150 μM) were added with PE Annexin V and 7-AAD and incubated for 15 min at room temperature in the dark, before flow cytometry analysis. The samples were run on the LSRFortessa flow cytometer (BD Biosciences, San Jose, CA) and analysed using the FlowJo (Tree Star Inc., Ashland, USA) analysis software. The apoptotic cells were also detected through TUNEL reaction by using terminal deoxynucleotidyl transferase assay kit (In Situ Cell Death Detection Kit, Roche Diagnostic, Basel, CH). Briefly, the cells were fixed with 4% paraformaldehyde for 20 min followed by the permeabilization with 0.1% Triton X-100 in 0.1% sodium citrate solution. After washing with PBS 1×, TUNEL reaction mixture was added and slides were then incubated for 60 min at 37 °C in the dark in a humidified atmosphere. TUNEL-positive cells were counted using fluorescence microscopy. The apoptotic cell number was scored by counting at least 5 fields of observation from three sample each group.

### 4.9. Chromatin Immunoprecipitation (ChIP) Assay

Dual crosslinking ChIP assay was used [32]. Briefly, cells were washed three times with room temperature PBS and PBS with 1mM MgCl_2_ was added. Disuccinimidyl Glutarate (DSG) (Sigma-Aldrich) was added to a final concentration of 2 mM for crosslinking and incubated 45 min at RT, formaldehyde (Sigma-Aldrich) was added to a final concentration of 1% (v/v) and cells incubated for 15 min for dual crosslinking, and glycine was added to a final concentration of 0.1 M and incubated for 10 min to quench formaldehyde cross-linking. After harvesting, cells were lysed in 500 μL ice-cold cell lysis buffer (50mM Tris/HCl pH8.0, 85 mM KCl, 0.5% NP40, 1mM PMSF, protease inhibitor cocktail) (Sigma-Aldrich) for 10 min on ice. Nuclei were precipitated by centrifugation (3,000g for 5 min), resuspended in 600µl ice-cold RIPA buffer (50 mM Tris/HCl pH 8.0, 150 mM NaCl, 1 mM EDTA pH 8.0, 1% Triton X-100, 0.1% SDS, 0.1% sodium deoxycolate, 1 mM PMSF, protease inhibitor cocktail) (Sigma-Aldrich) and incubated on ice for 30 min. Sonication was performed to obtain DNA fragments 100–600 bp in length using Soniprep 150 (MSE Sanyo, London, UK).

### 4.10. Mice Model and Treatments

NSG mice were bred under specific pathogen-free conditions in the Animal Facility of the University of Perugia. Eight to twelve-week-old male mice were used. Mice were subcutaneously injected into the right flank with 3 × 10^6^ A549 cells stably transfected with CTRL or PER1 plasmids. After tumors volume reached 50–80 mm^3^ mice were injected intraperitoneally with cisplatin (5mg/kg) every 3 days. Tumor size, expressed in mm^3^, was measured by a caliper for 15 days after tumor appearance and recorded every 2–3 days. Tumor volume was calculated as (width^2^ × length/2) and at sacrifice as length×width×height. Tumor weight (gr) was determined at sacrifice. Mouse experiments were performed according to Italian Approved Animal Welfare Authorization 669/2018-PR and Legislative Decree 26/2014 regarding the animal license obtained by the Italian Ministry of Health lasting for 2 years (2018–2020).

### 4.11. Histological Analysis

Tumors were removed and fixed in 10% phosphate-buffered formalin (Bio-Optica Milano Spa, Milan, Italy), embedded in paraffin and sectioned at 3 μm. For histological analysis, sections were stained with hematoxylin and eosin reagents. For TUNEL staining, sections were deparaffinized, rehydrated, and treated with 0.1 M citrate buffer, pH 6.0. The sections were then washed and blocked in 0.1 M Tris/HCl buffer, pH 7.5, supplemented with 3% bovine serum albumin and 20% FCS. The slides were then incubated with fluorescein-coupled dUTP and TUNEL enzyme (Roche Diagnostics) in the presence of terminal deoxynucleotidyltransferase. The samples were then washed with PBS. Nuclei were counterstained with DAPI. The sections were mounted and analyzed by fluorescent microscopy. Images were acquired using a BX51 microscope and analysis image processing software (Olympus, Tokyo, Japan).

### 4.12. Statistical Analysis

GraphPad Prism software 6.01 (GraphPad Software, Inc., San Diego, CA) was used for the analysis. Data are expressed as mean ± SD or SEM. Statistical significance was calculated by one- or two-way ANOVA (Tukey’s or Bonferroni’s post hoc test) for multiple comparisons and by a two-tailed Student’s t-test for single comparison. We considered all *p*-values < 0.05 significant. The in vivo groups consisted of 4 mice/group.
